# Trends in Antiretroviral Therapy and Prevalence of HIV Drug Resistance Mutations in Sweden 1997–2011

**DOI:** 10.1371/journal.pone.0059337

**Published:** 2013-03-22

**Authors:** Irene Bontell, Amanda Häggblom, Göran Bratt, Jan Albert, Anders Sönnerborg

**Affiliations:** 1 Department of Medicine, Karolinska Institutet Huddinge, Stockholm, Sweden; 2 Department of Clinical Science and Education, Venhälsan, Stockholm South General Hospital, Stockholm, Sweden; 3 Department of Virology, Swedish Institute for Infectious Disease Control, Solna, Sweden; 4 Department of Microbiology, Tumor and Cell Biology, Karolinska Institutet, Stockholm, Sweden; 5 Department of Laboratory Medicine, Virology, Karolinska Institutet Huddinge, Stockholm, Sweden; Alberta Provincial Laboratory for Public Health/University of Alberta, Canada

## Abstract

**Objective:**

Describe trends in antiretroviral treatments and drug resistance mutations among Swedish HIV-patients over time 1997–2011.

**Methods:**

Treatment histories, viral sequences, and demographic and clinical data were retrieved from the national database InfCareHIV. All ART-experienced patients were included (N = 6537), while resistance tests were restricted to those obtained ≥90 days after ART start. This cohort is fully representative for Sweden since the database covers virtually all diagnosed HIV-patients since the start of the epidemic. Patients were grouped according to the year of first ART, and treatments and mutations were analyzed by calendar year.

**Results:**

The prevalence of major drug resistance mutations decreased dramatically over time, most rapidly between 2003 and 2007. Since then there has been a continued slow decrease for NRTI- and PI-associated mutations with an overall prevalence among all ART-experienced patients at 1.1% (NRTI) and 0.3% (PI) in 2011. NNRTI resistance reached the lowest level in 2007–2009 (0.6%), but is now increasing (0.9% in 2011). Patients with first ART exposure before 2001 are still highly overrepresented among those with PI and, to a lesser extent, NRTI resistance. In contrast, almost half of the patients with NNRTI mutations in 2011 initiated their first ART after 2007.

**Conclusions:**

Tremendous improvements in ART options and knowledge have resulted in rapidly declining levels of resistance, and most of the current NRTI and PI mutations are found among patients with a history of suboptimal treatments. However, NNRTI resistance is increasing and is primarily found in patients infected in low- and middle-income countries who initiated ART in recent years. It is plausible that these patients were infected with resistant strains and it is therefore suggested that resource-rich countries like Sweden should test for resistance in minor quasispecies or use PI-based first-line regimens in patients who are at increased risk of carrying resistant virus.

## Introduction

In the early years of antiretroviral treatment (ART) only nucleoside analogue reverse transcriptase inhibitors (NRTIs) were available, which led to rapid development of drug resistance. In the mid-1990’s two new drug classes; non-nucleoside analogue reverse transcriptase inhibitors (NNRTIs) and protease inhibitors (PIs) were introduced, which soon led to effective combination therapies with improved clinical outcome and increased thresholds for development of drug resistance mutations (DRMs). The subsequent development of new drugs within the established drug classes and also drugs with new molecular targets has led to more treatment options, but drug resistance remains a concern and over 100 mutations that confer different levels of resistance to one or more antiretroviral drugs have been identified in the HIV-1 genome [1].

Several studies from the early years of 2000 showed an alarming prevalence of DRMs to the three main antiretroviral drug classes [2]–[4], but more recently a decreasing trend has been reported from many countries [5]–[9]. Sweden introduced routine genotypic resistance testing (GRT) at treatment failure as early as 1997, and the Swedish InfCare cohort (which includes all HIV-patients from the major cities since the beginning of the epidemic and all patients from the smaller clinics since 2007) is exceptional in terms of coverage, data quality and low frequency of missing data. The aim of this retrospective study is to describe the treatment patterns and drug resistance development in Swedish HIV-patients during the 15 years of GRT, 1997–2011, and to assess the current situation and identify challenges ahead.

## Methods

### Ethics Statement

The research has been approved by the Regional Ethical Review Board in Stockholm (2005/1167–31/3).

### Study Populations

All Swedish residents with known HIV infection are included in the national database and clinical decision support tool InfCareHIV (however, before 2007 some small clinics did not participate). InfCareHIV contains demographic and clinical data as well as treatment history and GRT results from routine testing, including viral sequences. A total of 8714 individuals had been registered in InfCareHIV up until 31^st^ Dec 2011, and 6537 (75.0%) of these had been exposed to ART and were included in the study. 1617 (24.7%) of the ART-experienced patients had a genotypic resistance test before start of therapy: the proportion of patients with baseline GRT increased from 2.1% in 1996 to 65.9% in 2011. Genotypic results from treatment naïve patients were excluded.

The treatment history of a patient strongly influences the development of DRMs and the number of antiretroviral drugs available. Treatment options and knowledge about how to use ART has improved greatly over time and therefore patients were subdivided into four groups according to their first ART start date: 1741 patients initiated ART in 1987–1996 (group I), 1181 between 1997 and 2001 (group II), 1434 between 2002 and 2006 (group III), while 2181 patients first used ART in the period 2007–2011 (group IV). Group I includes all patients who started ART prior to the introduction of GRTs in Sweden in 1997, while the three latter groups cover the consecutive five-year intervals during the study period.

The purpose of the study was to investigate the total prevalence of drug resistance over time and therefore all patients on treatment/all patients with a GRT in a given year were included in the denominator. That is, patients were not excluded after their first GRT (but only one GRT per patient per year was taken into account). This study design was selected as it best reflects the total prevalence of circulating mutations in the population and also shows the contribution of patients who have been on treatment for a very long time to the total level of DRMs.

### Analysis of Drug Resistance

GRTs from the years 1997–2011 from therapy experienced patients tested >90 days after first ART were included (2795 GRTs from 1548 individuals). If several tests were available from the same patient in one calendar year, only the latest one was included. Full sequence information was unavailable for 301 GRTs (10.8%) instead a mutation list was used. Major resistance mutations were defined according to the IAS-USA 2011 list (1). The level of clinically relevant resistance for each drug was determined using the Stanford HIVdb algorithm [10] and the level of resistance was retrieved for seven NRTIs (3TC, ABC, AZT, d4T, ddI, FTC and TDF), four NNRTIs (EFV, NVP, ETR and RPV) and eight PIs (ATV/r, DRV/r, FPV/r, IDV/r, LPV/r, NFV, SQV/r and TPV/r). A drug was considered “effective” if the virus carried no or low-level resistance to it, while it was considered “ineffective” if the virus had intermediate or high-level resistance the drug. Full-class resistance (FCR) was defined as intermediate or high-level resistance to all drugs in the class. Furthermore, exhaustion of available drug options was investigated and for this analysis, only drugs that were in use in Sweden at the time were included (1997∶3TC, ZDV, d4T, ddI, ddC, FPV, IDV, NFV, SQV, RTV, NVP; 1998: EFV; 2000: LPV; 2001: T-20; 2002: TDF; 2003 ATV, TPV; 2005: FTC; 2006: DRV, RAL; 2007: ETR; 2008: MVC; 2011: RPV). Since most ART-combinations consist of a backbone of 2 NRTIs +1 PI/r or 1 NNRTI, the possibility to effectively use this kind of triple therapy was assessed separately. A NRTI backbone was considered available only if two compatible NRTIs were found to be effective (3TC+FTC, ddI+d4T, d4T+AZT and ddI+TDF are not considered acceptable combinations for a NRTI backbone according to Swedish guidelines) NRTI wide-class resistance (WCR) was thus defined as high-level or intermediate resistance to all NRTIs except one, or two incompatible ones.

For statistical analysis of categorical variables chi-square tests were used and for numerical variables t-test.

## Results and Discussion

### Patient Characteristics

Substantial demographic changes among Swedish HIV patients can be seen over time in the database. The proportions of homosexually infected men and intravenous drug users have decreased while there has been an increase in the number of women and heterosexually infected patients. The proportion of patients born and infected abroad has also increased, with large patients groups coming from sub-Saharan Africa and South-East Asia. This has also led to an increase of patients infected with non-B subtypes, especially subtype C and CRF01_AE. Details of patient characteristics for the four groups I–IV is shown in Table 1.

**Table 1:** Patient characteristics of Groups I–IV.

	Group I (N = 1741),first ART 1987–1996	Group 2 (N = 1181), first ART 1997–2001	Group III (N = 1434), first ART 2002–2006	Group IV (N = 2181), first ART 2007–2011	Total (N = 6537)
Women	20.4%	36.5%	42.1%	38.6%	**34.2%**
Age at first ART	37.8±10.1	37.6±11.2	38.3±11.9	38.9±12.3	**38.2±11.5**
Born in Sweden	55.8%	47.8%	34.2%	34.9%	**42.6%**
Infected in Sweden	61.2%	44.9%	35.4%	38.7%	**45.1%**
Heterosexual	28.0%	48.6%	58.4%	50.7%	**46.0%**
Homosexual	51.6%	30.3%	23.2%	27.5%	**33.5%**
IDU	12.5%	10.3%	7.8%	7.0%	**9.3%**
Subtype B*	77.2%	58.2%	43.4%	40.4%	**52.6%**

* per cent of infections with known subtype that were caused by HIV-1 B.

### Total ART-exposure of Swedish HIV-patients

**Figure 1:**
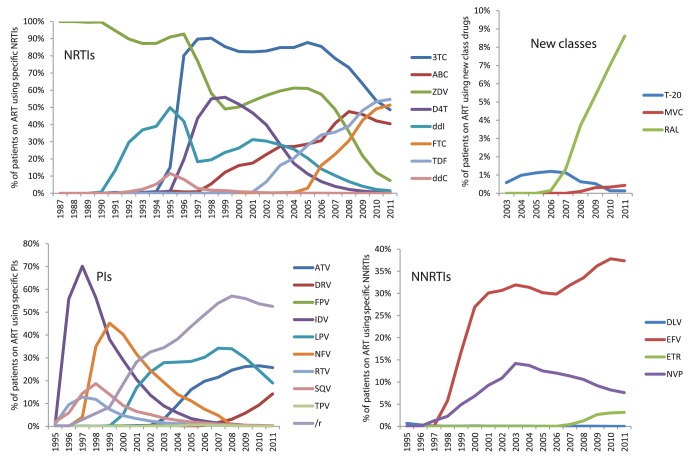
Antiretroviral drug use in Sweden 1987–2011. The graphs depict the proportion of patients on ART exposed to each individual drug per calendar year, revealing trends in usage prevalence overtime.

The mean time on first line treatment before switch or treatment cessation was 16.6 months (all additions or removals of drugs were counted as a switch), and the average study participant had had 4.3 treatments and been exposed to 6.2 different drugs by 31^st^ Dec 2011. Out of all the 6537 patients, 6519 (99.7%) had been exposed to NRTIs, 3792 (58.0%) to NNRTIs and 4666 (71.4%) to PIs. Among the newer classes, the integrase inhibitor (II) Raltegravir was most commonly used (511 patients, 7.8%), while fusion inhibitors (FI) and entry inhibitors (EI) had only been used by 86 (1.3%) and 34 (0.5%) patients respectively. As expected, there were substantial differences between the four groups I–IV, as shown in Table 2, where patients with an early ART start had experienced a larger number of drugs. Figure 1 provides detailed information on the yearly prevalence of each individual drug within the population on ART, where several trends are clearly visible: the replacement of first generation PIs by LPV/r from 2000 onwards and later by ATV/r and DRV/r; the increased use of NNRTIs over time, especially EFV; the gradual phasing out of ZDV and several other NRTIs leaving four main drugs to construct the backbone of most modern therapies: FTC, TDF, 3TC and ABC; and finally the steady rise of RAL over the past five years, while other new class drugs have been used very sparsely in Sweden so far.

**Table 2:** Drug exposure among the four patient groups I–IV.

	N	Still on ART^1^	Still on first line ART^1^	Average number of treatments	Average number of drugs	NRTI exposure	PI exposure	PI boost exposure	NNRTI exposure	II exposure	FI exposure	EI exposure
Group I	1741	53%	0%	7.2	7.4	100.0%	68.6%	48.7%	51.8%	11.1%	3.2%	0.9%
Group II	1181	81%	2%	5.6	7.6	100.0%	87.1%	62.7%	67.7%	8.2%	1.0%	0.5%
Group III	1434	89%	8%	3.7	6.2	99.9%	77.4%	74.4%	61.9%	5.0%	0.6%	0.3%
Group IV	2181	93%	52%	1.8	4.4	99.2%	61.1%	60.4%	55.1%	6.8%	0.4%	0.3%
Total	**6537**	**79%**	**20%**	**4.3**	**6.2**	**99.7%**	**71.4%**	**60.8%**	**58.0%**	**7.8%**	**1.3%**	**0.5%**

1 as of 2011–12–31.

### Changes in First Line ART Over Time

**Figure 2:**
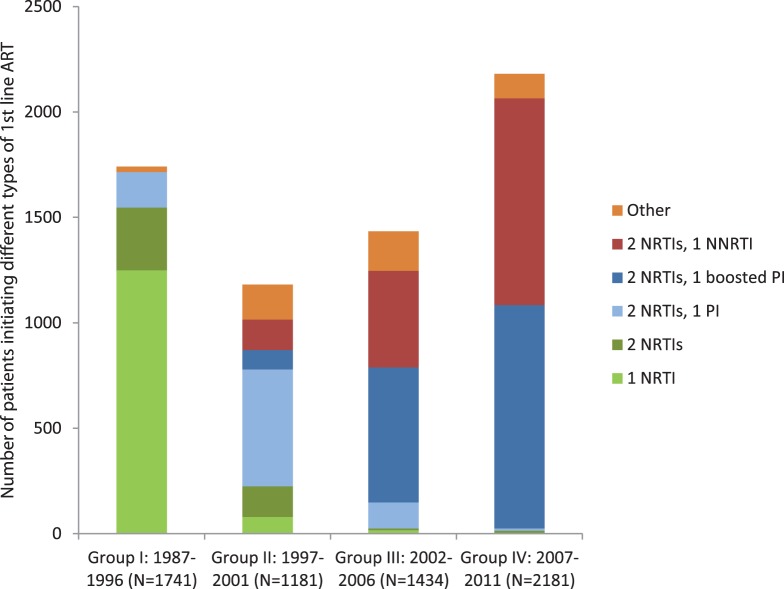
Differences in first line ART combinations over time. The coloured bars show the total number of patients in groups I–IV who initiated different kinds of first line ART: 1 NRTI only; 2 NRTIs; 2 NRTIs +1 (unboosted) PI, 2 NRTIs +1 boosted PI, 2 NRTIs +1 NNRTI or Other combination (for example 3 NRTIs, 3 NRTIs +1 PI or 1 NNRTI, 1 NRTI +1 PI +1 NNRTI or combinations including new drug classes).

More than two thirds of all patients starting ART in Sweden between 1987 and 1996 were given the same therapy – ZDV monotherapy. In the following five-year period a much greater diversity was seen; a total of 110 different combinations were prescribed as first-line therapy in the period 1997–2001, most commonly 2 NRTIs (3TC+ZDV or d4T) plus an unboosted PI (IDV or NFV), but ZDV monotherapy was still initiated in 5.9% of all patients. During the next period, 2002–2006, the NRTI backbone 3TC+ZDV was predominant (66.2%), mostly in combination with boosted LPV or an NNRTI (EFV or NVP). In the most recent five-year period (2007–2011), the most common NRTI backbones were FTC+TDF (51.1%) and 3TC+ABC (30.7%), most often combined with EFV or a boosted PI (LPV/r or ATV/r). Figure 2 provides an overview of the type of first line ART in Sweden over time, while Table S1 details the 10 most common first line combinations for each time period.

### Prevalence of Major Drug Resistance Mutations Over Time

**Figure 3:**
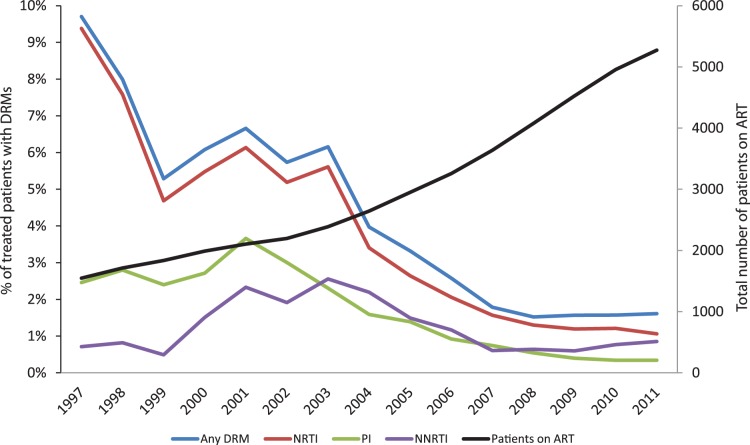
Prevalence of major resistance mutations among all ART-experienced patients 1997–2011. The coloured graphs show the proportion of patients on treatment each year that had at least one major drug resistance mutation in that year. Any DRM includes all patients with at least one major resistance mutation to any drug class while separate graphs show the prevalence of ≥1 mutation associated with resistance to NRTI, NNRTI and PI respectively. The black line illustrates the total number of Swedish HIV-patients on ART each calendar year.

Although the number of patients on ART has increased steadily, from N = 1546 in 1997 to N = 5272 in 2011, the number of GRTs at treatment failure has only showed relatively small fluctuations with an average of 186 tests per year (Table S2). As shown in Figure 3,the prevalence of mutations associated with resistance to different drug classes exhibit different trends over time: NRTI associated DRMs have decreased from a total prevalence of 9.4% in the population on ART in 1997 (corresponding to 68.5% of patients with a GRT) to 1.06% in 2011 (39.4% of GRTs). PI mutations were found at the highest prevalence in 2001∶3.7% of patients on ART (33.6% of GRTs), decreasing to 0.34% in 2011 (8.3% of GRTs). NNRTI mutations reached a peak in 2003–2004 (2.6% of patients on ART (2003), 37.7% of GRTs (2004)) followed by a decrease to 0.60–0.64% of patients on ART in 2007–2009 and thereafter an increase to 0.77% in 2010 and 0.85% in 2011. Resistance to new class drugs is still very limited and does not contribute significantly to the overall resistance trend; the maximum number so far was detected in 2011 with four cases of resistance towards raltegravir. A full overview of the number of patients, GRTs and prevalence of DRMs over the years is provided in Table S2.

**Figure 4:**
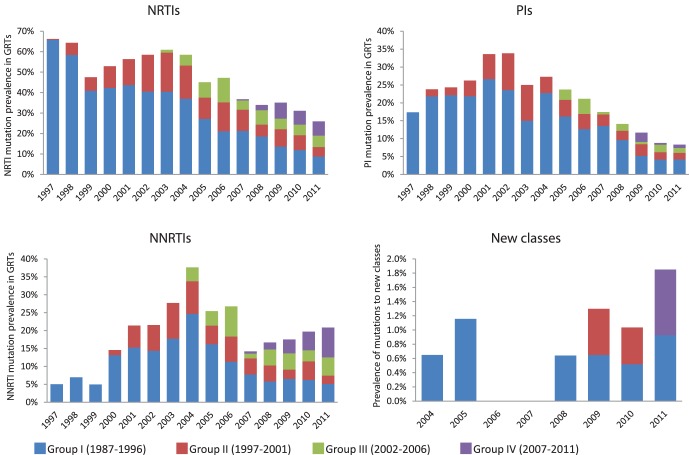
Resistance by year of first ART. These graphs show the level of resistance in each given year by the four groups, I–IV. This aims to show the composition of resistance over time, where patients with early exposure to ART (groups I and II) constitute the vast majority of resistance cases. Even in recent years, most resistance is found among these patients although they comprise a relatively small portion of the total number of patients on ART (group I patients corresponded to just 18% of patients on ART in 2011). Important differences can be noted between drug classes, where group IV patients contribute to a substantial proportion of recent NNRTI mutations while hardly any PI mutations have been found in this group.

The DRM results were further analyzed by group I–IV (Figure 4). These results show that a large majority of all patients with resistance in the period 1997–2011 had first been exposed to ART before 1997. Over the entire 15 year period, 69.8% of all GRTs with NRTI mutations came from group I patients and the corresponding figures were 75.2% for PI, 58.2% for NNRTI and 66.7% for new class drugs. This is not surprising seen over the whole period, since these patients have been on ART for a very long time and been exposed to suboptimal therapies in the past and also comprise 53.3% of all GRTs included in the study. However, even in 2011 when group I patients constituted only 17.9% of the patients on ART (18.1% of GRTs) they still represented 33.9% and 50% of the NRTI and PI resistant populations respectively, significantly higher than expected (P<0.005, chi-square test). This pattern was not seen for NNRTI, where the observed number of resistant patients from group I was not significantly higher than the expected number (P = 0.3, chi-square test). Instead, NNRTI mutations have increased substantially in group IV patients (initiating their first ART in 2007–2011) in recent years. This can partly be explained by the large number of patients in group IV; 39.4% of patients on ART and 40.3% of patients with GRT in 2011. The observed number of group IV patients with NNRTI mutations in 2011 equals the expected number (P = 0.97) if all groups have the same likelihood of resistance development. Nonetheless, this continuous development of DRMs in patients starting ART since 2007 sets NNRTI apart from the other historical drug classes, where there is a decreasing trend; PI resistance for group IV in 2011 was significantly lower than expected (P = 0.04, chi-square test), and the same trend was seen for NRTI, although with borderline significance (P = 0.06, chi-square test).

**Figure 5:**
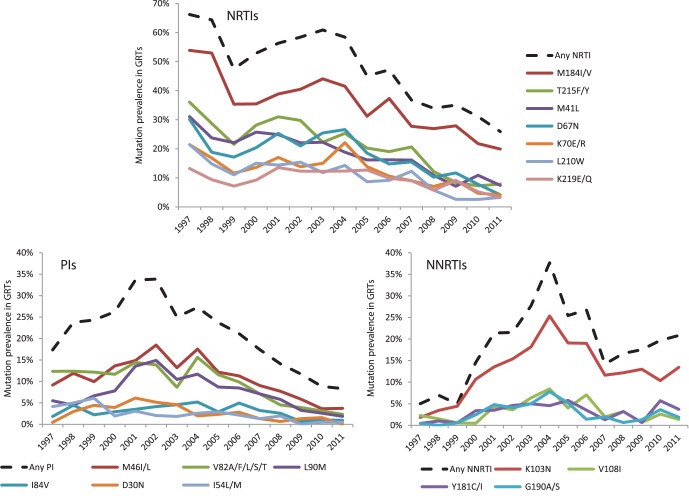
Prevalence trends of individual resistance mutations 1997–2011. Prevalence of the most common resistance mutations in relation to all performed GRTs per calendar year. All NRTI, PI and NNRTI associated mutations with a prevalence of at least 5% of GRTs any year have been included.

The prevalence trends of individual mutations were also investigated (Figure 5). The most common major drug resistance mutation throughout the entire 15-year period was M184V/I, found in 36.2% of all GRTs and present in three out of four (74.4%) patients with NRTI resistance. The thymidine analogue mutations (TAMs) also had high prevalences; T215F/Y (21.8%), M41L (18.9%), D67N (18.2%), K70E/R (12.8%), L210W (11.1%) and K219E/Q (9.7%). All of these exhibited decreasing trends over time, where the most rapid decline was seen after 2004. Other NRTI DRMs had an overall prevalence below 2% (in decreasing order: L74V, F116V, K65R, A62V, Y115F, V75I, Q151M and F77L) and no trends over time could be determined for these as the total number of detected mutations was very small. Among NNRTI DRMs, K103N was the predominant one, present in 2 out of 3 (67.2%) patients with NNRTI mutations, and with a total prevalence of 12.4% of all GRTs). The K103N prevalence showed a steep increase until a peak at 25.3% in 2004, followed by a sharp decline until 2007 where it plateaus out around 12% of all GRTs. The prevalence of the other main NNRTI mutations, V108I (3.1%), Y181C/I) 3.1% and G190A/S (2.8%), also peaked in 2004 or 2005, albeit less sharply. Common PI DRMs were M46I/L (10.8%), V82A/F/L/S/T (9.7%), L90M (7.5%), I84V (3.0%), D30N (2.7%) and I54L/M (2.4%), most of which had a peak prevalence around 2002, with a significant decline from 2004–2011. G48V, V32I, N88S, T74P, I47A/V, Q58E, I50L/V, L76V and N83D had an overall prevalence below 2% and did not show any trends over time.

### M184I/V and K103N

The most common mutations for NRTI and NNRTI, M184I/V and K103N, are responsible for a large proportion of the total resistance problem and are major contributors to the overall trends. M184I/V was present in 74.4% of all patients with any major NRTI mutation, and the corresponding figure for K103N was 67.2%, and these proportions remained all but constant over time.

**Figure 6:**
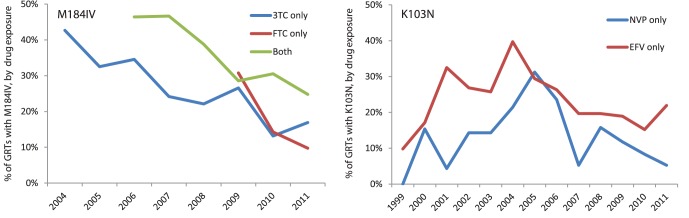
Prevalence of M184IV and K103N by drug exposure. The prevalence of M184IV was divided into patients who had been exposed to 3TC but not FTC (3TC only), FTC but not 3TC (FTC only); or both 3TC and FTC (the graphs show % of GRTs on patients from each group that contained the mutation). No difference could be detected between 3TC only and FTC only, while the patients with exposure to both drugs were more likely to carry resistant virus. In the corresponding graph for K103N it is shown that patients exposed to EFV consistently had higher mutation prevalence compared to patients with NVP. The limited number of patients with exposure to both EFV and NVP had intermediate values and are omitted for clarity.

The M184IV mutation is selected by and confers resistance to 3TC and FTC, which are present in the NRTI backbones of nearly all ART regimens. The use of FTC increased rapidly between 2005 (N = 92) and 2011 (N = 2827), while the use of 3TC has started to decline in recent years (2008: N = 2913, 2011∶2443). However, most patients on FTC have previously been exposed to 3TC. In order to see whether the choice of cytosine analogue affects the risk of M184IV development, all GRT dates were compared with the dates of 3TC and FTC initiation and each resistance test classified as “3TC only”, “FTC only”, or “Both” if the patient had been exposed to both 3TC and FTC. As expected, the prevalence of M184IV was highest among patients who had been exposed to both 3TC and FTC (Figure 6) and the difference was significant compared to patients who had been exposed to 3TC only (P = 0.02, t-test). There was no obvious difference in the M184IV prevalence between 3TC and FTC exposed patients and since the total number of GRTs on patients exposed to FTC only was very small (ranging from only 2 in 2005–2007 to 41 in 2011) and the GRTs positive for M184IV even smaller (between 0 and 4), it is too early to draw any conclusions on differences in M184IV development between 3TC and FTC based on this cohort.

K103N causes resistance to NVP and EFV. As shown in Figure 1, EFV has been used much more frequently in Sweden and is recommended over NVP by national and international guidelines. In 2011, the total number of people on EFV was 1969, compared to 401 on NVP. When the same process as for M184IV was applied to K103N, the results showed that patients exposed to EFV was significantly more likely to harbour virus with the K103N mutation compared to those exposed to NVP (P = 0.005, t-test; Figure 6). The relatively small number of patients exposed to both drugs had intermediate levels of resistance (data not shown).

A recent study [11] has shown that out of five common 3TC/FTC-based ART regimens, the combination of EFV and ZDV resulted in the highest risk of resistance development. This type of combination is decreasing in Sweden: 3TC+ZDV+EFV was the second most commonly prescribed first line combination in 2002–2006, but was only used as first line ART by 1.7% in 2007–2011 (Table S1). Out of the 29 patients with K103N in Sweden in 2011, only two had been exposed to this treatment. It has, however, been the preferred ART combination for a long time in many low- and middle income countries.

The large proportion of patients with NNRTI mutations who had a recent ART start (Figure 4) is of great concern. K103N mutated virus was found in a total of 79 individuals in 2007–2011, 60 of whom had been exposed to EFV only. Nearly half (12/25) of the EFV exposed patients with K103N in 2011 initiated first line ART in the past five years and 9/25 had been on ART ≤2 years. The reason for this high level of early failures with K103N is not known and poor adherence is a possibility. It is noteworthy that a large majority of these patients (18/25) were infected in low-and middle income countries and none of the 9 cases with ≤2 years ART had been acquired in Sweden. It is possible that some patients carried virus with transmitted resistance that was present at levels too low for detection by the standard genotyping procedures preceding ART initiation (notably, no baseline GRT had been performed for two of the patients initiating ART in 2009, and it is of utmost importance that this is routinely performed at all clinics). The risk of undetectable transmitted resistance should be considered when initiating treatment, and PI-based regimens may be preferable as first line ART in patients at increased risk of harbouring resistant virus.

### Full-class Resistance and Exhaustion of Treatment Options

**Figure 7:**
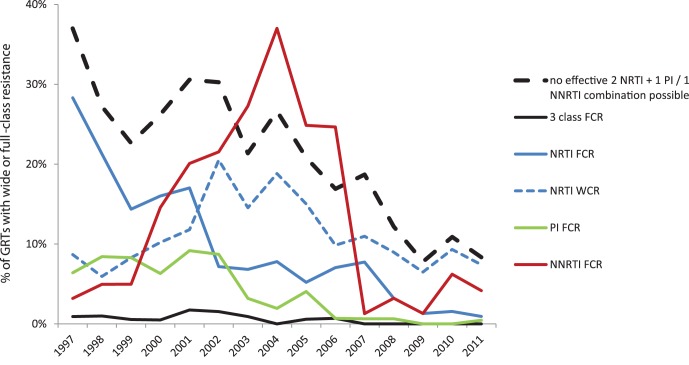
Full-class resistance to one or several classes 1997–2011. Percentage of GRTs containing virus that is fully resistant to one or more drug classes. Full-class resistance (FCR) was defined as intermediate or high-level resistance to all drugs in the class that were available in Sweden that year. In addition, for NRTI, wide-class resistance (WCR) prohibiting the use of an effective 2 NRTI backbone is shown (blue dashed line). The black dashed line shows total resistance prohibiting efficient use of any standard regimen (2 compatible NRTIs +1 PI or 1 NNRTI).

Full-class resistance (FCR), defined as intermediate or high-level resistance to all available drugs in a class, and exhaustion of treatment options is depicted in Figure 7. NRTI-FCR was initially high, over 15% of GRTs until 2001, but declined sharply in 2002 with the introduction of TDF, and has levelled off around 1–1.5% of patients with GRTs in the past three years. However, in order to construct an effective NRTI backbone, the virus must be susceptible to at least two compatible NRTIs. The proportion of GRTs with wide-class NRTI resistance prohibiting effective combination therapy remains substantial with levels around 7–9% over the past four years. PI-FCR showed a remarkable decrease over time, from close to 1 in 10 GRTs in 2001 to zero eight years later. The introduction of ATV/r and TPV/r in 2003 and subsequently DRV/r in 2006 have practically eliminated FCR for this class. Between 2006 and 2011, PI-FCR was only detected in four patients in Sweden, all of whom had their first ART before 1997. NNRTI-FCR, on the other hand, rose sharply to a peak prevalence of 37.0% in 2004, which is very close to the prevalence of any major NNRTI mutation this year (37.7% of all GRTs). This was a consequence of the cross-resistance between the only available NNRTIs at the time, where a single mutation could be enough for full-class resistance. Since the introduction of ETR in 2007 NNRTI-FCR has been less of a problem, but it is still high compared to NRTI- and PI-FCR.

In total, the proportion of patients for whom there was no available combination of a functional NRTI-backbone plus at least one PI/r or NNRTI has decreased from 37.0% of patients with GRT in 1997 to 8.3% in 2011, which corresponds to just 18 patients. Since there are also additional alternatives with new class drugs there are few, if any, HIV-patients in Sweden for whom no treatment is likely to be effective.

### Conclusions

This study describes antiretroviral treatment and associated drug resistance mutations in Swedish HIV-patients over time and shows how out-phasing of older drugs has been followed by a decreasing prevalence of associated mutations. Better understanding of resistance has resulted in improved guidelines and quality of care and the wide range of current antiretroviral drugs mean that effective options are generally available even for highly treatment experienced patients.

However, a disturbing break of the overall decreasing resistance trends is the recent increase in NNRTI mutations, which may be a result of undetected transmitted mutations in patients infected in endemic countries. The massive ART roll-out in low- and middle income countries, which is mostly NNRTI based, save millions of lives but can also lead to transmission of resistant viruses. Although viral genotyping should always be performed prior to ART initiation in Sweden, minor populations of resistant virus may not be detected by standard methods. Resource-rich settings like Sweden should therefore consider recommending PI-based regimens as the first choice in patients infected in low- and middle income countries or perform quantitative PCR or deep sequencing for detection of resistance in minor quasispecies prior to initiation of NNRTI treatment.

## Supporting Information

Table S1 **The 10 most common first line ART regimens in Sweden during different periods of time.**
(DOCX)

Table S2 **Total numbers of patients on ART, with GRT and major DRMs per year 1997–2011, and the prevalence of patients with different types of DRMs in relation to the all the patients on treatment that year.**
(DOCX)
