# Supplementary material for: Trends in Antiretroviral Therapy and Prevalence of HIV Drug Resistance Mutations in Sweden 1997–2011
Source: PLoS One. 2013 Mar 22;8(3):e59337. doi: 10.1371/journal.pone.0059337 (PMC3606434; doi:10.1371/journal.pone.0059337)
Supplement: Table S1 — The 10 most common first line ART regimens in Sweden during different periods of time. (DOCX) [file pone.0059337.s001.docx]

**Table S1.** The 10 most common first line ART regimens in Sweden during different periods of time (% of all first line ART prescribed during that period).

|  | **Group I: 1987-1996, N=1741** | **Group II: 1997-2001, N=1181** | **Group III: 2002-2006, N=1434** | **Group IV: 2007-2011, N=2181** |
| --- | --- | --- | --- | --- |
| 1 | ZDV (**67.4%**) | 3TC, ZDV, IDV (**17.9%**) | 3TC, ZDV, LPV/r (**29.1%**) | FTC, TDF, EFV (**26.9%**) |
| 2 | ZDV, ddI (**8.8%**) | 3TC, ZDV, NFV (**10.8%**) | 3TC, ZDV, EFV (**14.6%**) | 3TC, ABC, EFV (**11.8%**) |
| 3 | 3TC, ZDV, IDV (**7.8%**) | 3TC, d4T, NFV (**8.5%**) | 3TC, ZDV, NFV (**5.8%**) | 3TC, ZDV, LPV/r (**9.6%**) |
| 4 | 3TC, ZDV (**6.9%**) | 3TC, ZDV (**7.8%**) | 3TC, ZDV, NVP (**4.0%**) | FTC, TDF, ATV/r (**9.1%**) |
| 5 | ddI (**3.1%**) | 3TC, ZDV, EFV (**6.3%**) | 3TC, ABC, ZDV (**3.2%**) | 3TC, ABC, LPV/r (**8.3%**) |
| 6 | ZDV, ddC (**0.7%**) | ZDV (**5.9%**) | 3TC, TDF, EFV (**3.0%**) | FTC, TDF, LPV/r (**7.8%**) |
| 7 | ABC (**0.7%**) | 3TC, d4T, IDV(**4.5%**) | FTC, TDF, EFV (**2.8%**) | 3TC, ABC, ATV/r (**7.3%**) |
| 8 | 3TC, ZDV, RTV (**0.5%**) | 3TC, ZDV, LPV/r (**2.8%**) | 3TC, TDF, ATV/r (**2.7%**) | FTC, TDF, DRV/r (**4.0%**) |
| 9 | 3TC (**0.5%**) | 3TC, ZDV, IDV/r (**2.5%**) | 3TC, d4T, NVP (**2.6%**) | 3TC, ZDV, EFV (**1.7%**) |
| 10 | 3TC, d4T (**0.4%**) | 3TC, ABC, ZDV (**1.9%**) | 3TC, ABC, LPV/r (**2.1%**) | 3TC, ZDV, NVP (**1.6%**) |
