# Supplementary material for: Trends in Antiretroviral Therapy and Prevalence of HIV Drug Resistance Mutations in Sweden 1997–2011
Source: PLoS One. 2013 Mar 22;8(3):e59337. doi: 10.1371/journal.pone.0059337 (PMC3606434; doi:10.1371/journal.pone.0059337)
Supplement: Table S2 — Total numbers of patients on ART, with GRT and major DRMs per year 1997–2011, and the prevalence of patients with different types of DRMs in relation to the all the patients on treatment that year. (DOCX) [file pone.0059337.s002.docx]

**Table S2.** Total numbers of patients on ART, with GRT and major DRMs per year 1997-2011, and the prevalence of patients with different types of DRMs in relation to the all the patients on treatment that year. The highest prevalence for each type of DRM is marked in regular bold script, while the lowest prevalence is in bold italics.

| **Year** | **Patients on ART** | **Patients with GRT** | **≥1 major DRM to any class** | **NRTI DRM** | **PI DRM** | **NNRTI DRM** | **2 class DRM** | **3 class DRM** | **FI or II DRM** |
| --- | --- | --- | --- | --- | --- | --- | --- | --- | --- |
| 1997 | 1546 | 219 | 150 **(9.70%)** | 145 **(9.38%)** | 38 (2.46%) | 11 (0.71%) | 41 (2.65%) | 4 (0.26%) |  |
| 1998 | 1714 | 202 | 137 (7.99%) | 130 (7.58%) | 48 (2.80%) | 14 (0.82%) | 48 (2.80%) | 8 (0.47%) |  |
| 1999 | 1835 | 181 | 97 (5.29%) | 86 (4.69%) | 44 (2.40%) | 9 ***(0.49%)*** | 39 (2.13%) | 6 (0.33%) |  |
| 2000 | 1990 | 206 | 121 (6.08%) | 109 (5.48%) | 54 (2.71%) | 30 (1.51%) | 62 (3.12%) | 14 (0.70%) |  |
| 2001 | 2103 | 229 | 140 (6.66%) | 129 (6.13%) | 77 **(3.66%)** | 49 (2.33%) | 87 **(4.14%)** | 31 **(1.47%)** |  |
| 2002 | 2197 | 195 | 126 (5.74%) | 114 (5.19%) | 66 (3.00%) | 42 (1.91%) | 73 (3.32%) | 23 (1.05%) |  |
| 2003 | 2388 | 220 | 147 (6.16%) | 134 (5.61%) | 55 (2.30%) | 61 **(2.55%)** | 84 (3.52%) | 19 (0.80%) |  |
| 2004 | 2643 | 154 | 105 (3.97%) | 90 (3.41%) | 42 (1.59%) | 58 (2.19%) | 66 (2.50%) | 20 (0.76%) | 1 (0.038%) |
| 2005 | 2951 | 173 | 98 (3.32%) | 78 (2.64%) | 41 (1.39%) | 44 (1.49%) | 49 (1.66%) | 18 (0.61%) | 2 (0.068%) |
| 2006 | 3257 | 142 | 84 (2.58%) | 67 (2.06%) | 30 (0.92%) | 38 (1.17%) | 39 (1.20%) | 13 (0.40%) | 0 ***(0.000%)*** |
| 2007 | 3638 | 155 | 65 (1.79%) | 57 (1.57%) | 27 (0.74%) | 22 (0.60%) | 35 (0.96%) | 7 (0.19%) | 0 ***(0.000%)*** |
| 2008 | 4077 | 156 | 62 ***(1.52%)*** | 53 (1.30%) | 22 (0.54%) | 26 (0.64%) | 32 (0.78%) | 7 (0.17%) | 1 (0.025%) |
| 2009 | 4526 | 154 | 71 (1.57%) | 54 (1.19%) | 18 (0.40%) | 27 (0.60%) | 25 ***(0.55%)*** | 4 (0.09%) | 2 (0.044%) |
| 2010 | 4956 | 193 | 78 (1.57%) | 60 (1.21%) | 17 ***(0.34%)*** | 38 (0.77%) | 31 (0.63%) | 6 (0.12%) | 2 (0.040%) |
| 2011 | 5272 | 216 | 85 (1.61%) | 56 ***(1.06%)*** | 18 ***(0.34%)*** | 45 (0.85%) | 33 (0.63%) | 3 ***(0.06%)*** | 4 **(0.076%)** |
